# Supplementary material for: Development of a Human B7-H3-Specific Antibody with Activity against Colorectal Cancer Cells through a Synthetic Nanobody Library
Source: Bioengineering (Basel). 2024 Apr 15;11(4):381. doi: 10.3390/bioengineering11040381 (PMC11047927; doi:10.3390/bioengineering11040381)
Supplement: Supplementary file 1 [file bioengineering-11-00381-s001.zip › bioengineering-2941329-supplementary.pdf]

Figure S1. BLI sensorgrams revealed that A6 and a13 did not bind to murine B7-H3. The red lines indicated the transition from the association step (300 s) to dissociation step (600 s) in the BLI experiment. A serial 3-fold dilution of these antibodies were used in this experiment with 2.7 μM as the highest concentration tested.

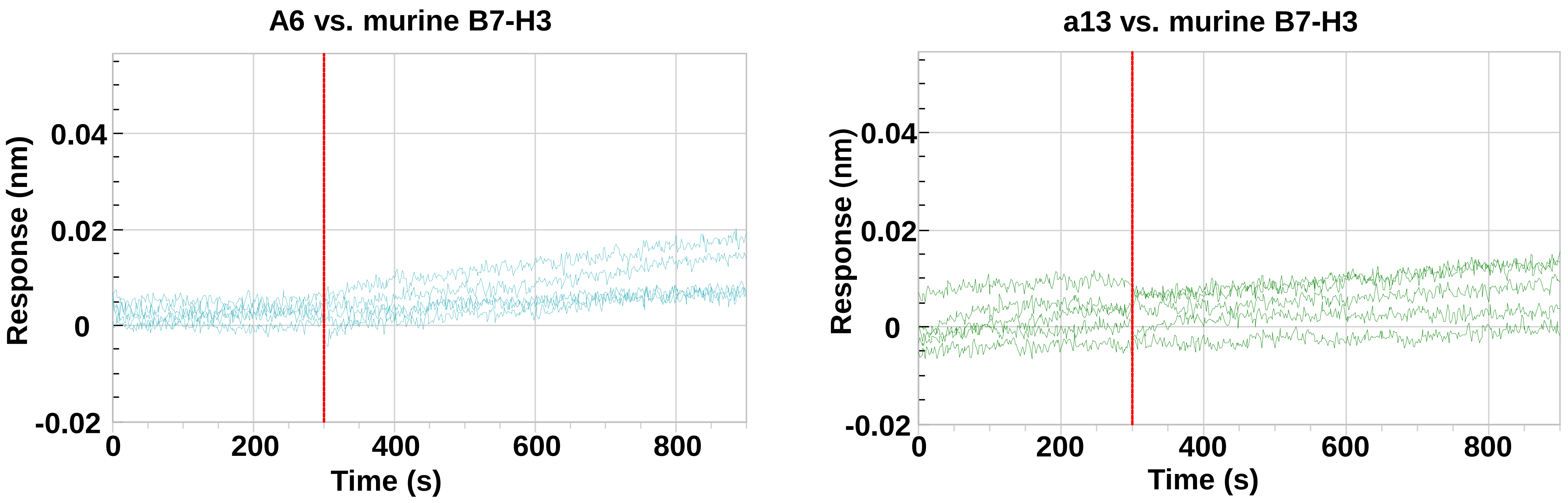

Figure S2. Single dose BLI sensorgrams revealed that A6 and 8H9 bound to C-2IgB7-H3 and N-2IgB7-H3 similarly. The red lines indicated the transition from the association step (420 s) to dissociation step (580 s) in the BLI experiment. Sensorgrams of A6 was colored cyan, and those of 8H9 colored blue.

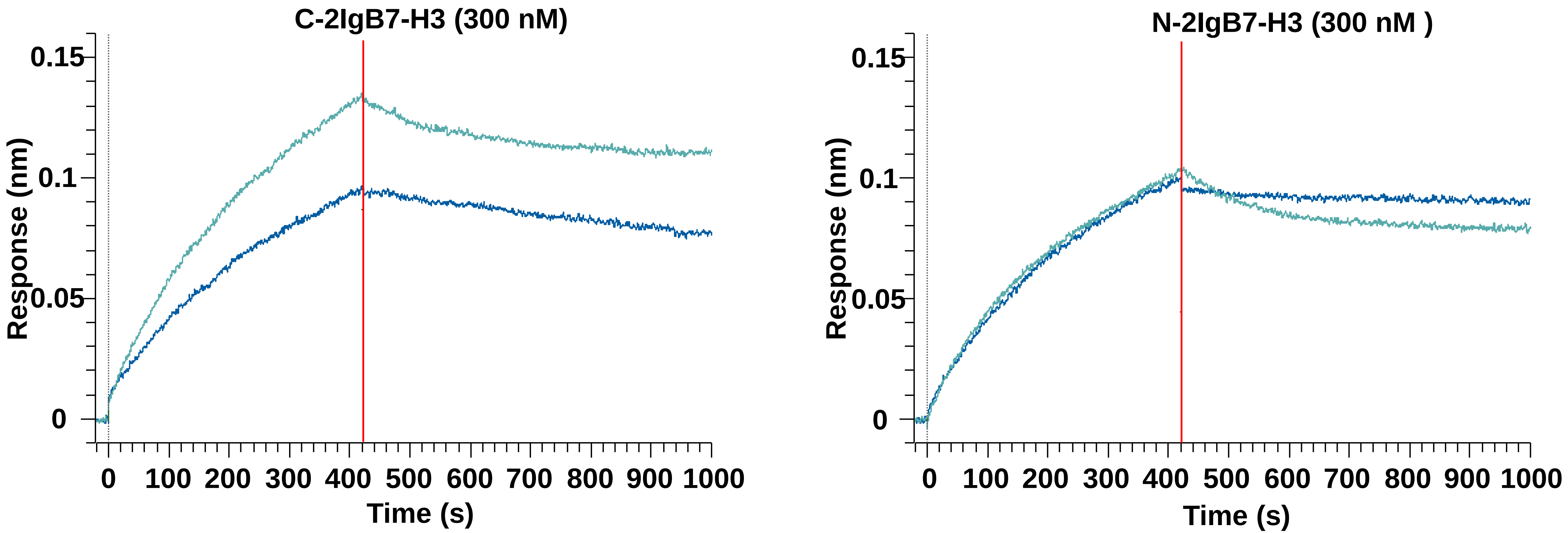

Figure S3. The complete sensorgrams of the epitope binning experiment. (A) 8H9 was used as immobilized MAb1, and each of A6 and a13 as sandwishing MAb2. 8H9 was used as an control for full epitope overlap. (B) A6 was used as immobilized MAb1, and a13 as sandwishing MAb2. A6 was used as an control for full epitope overlap.

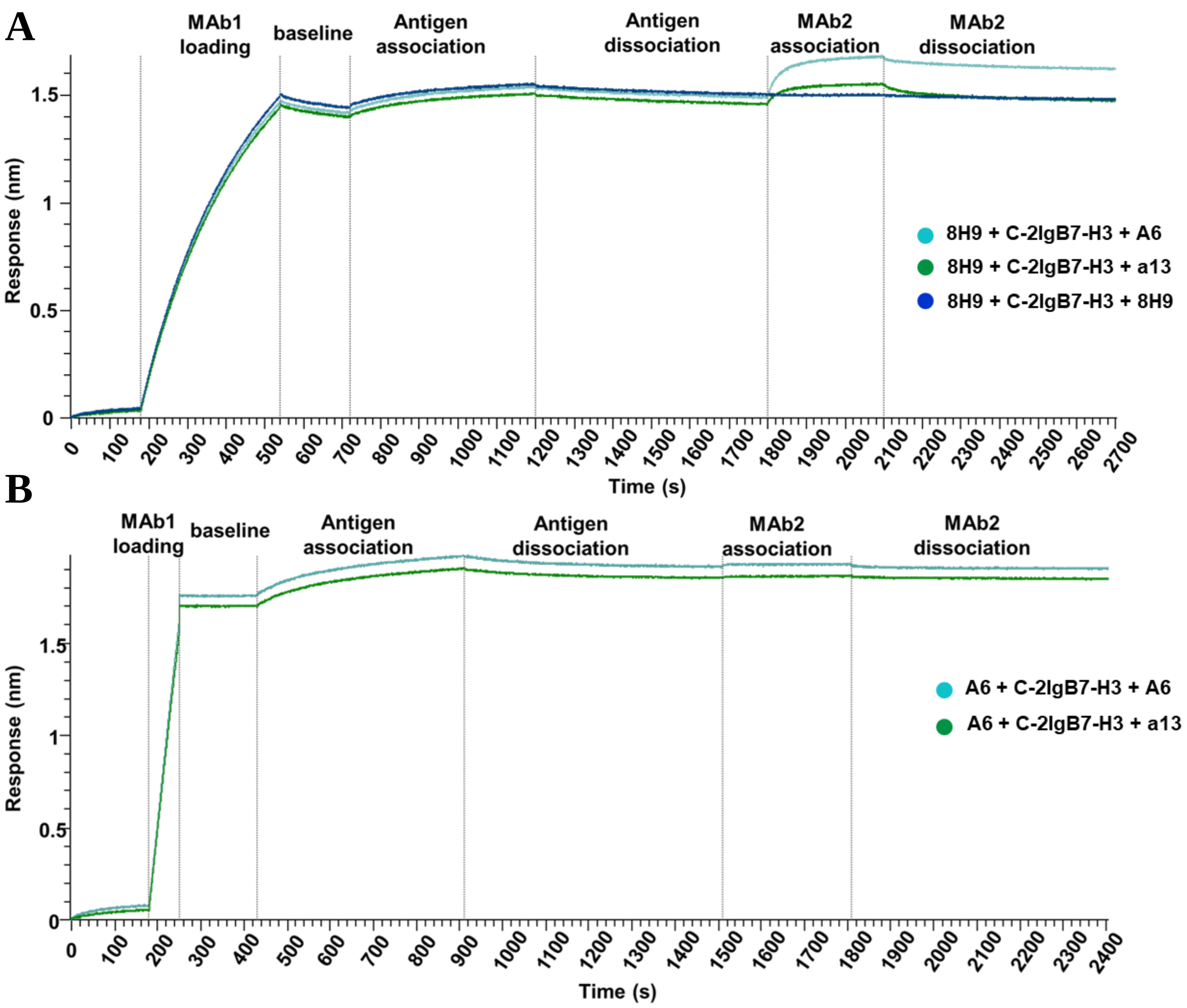

Figure S4. Generation of a B7-H3 knockdown HCT116 cell pool. The expression level of B7-H3 was detected by anti-B7-H3 rabbit polyclonal antibody as the primary antibody. GAPDH was used as the loading control. Jurkat and Expi293F were used as negative and positive control for B7-H3 expression, respectively. The level of B7-H3 expression in the B7-H3 knockdown HCT116 cell pools are significantly lower than HCT116 cells.

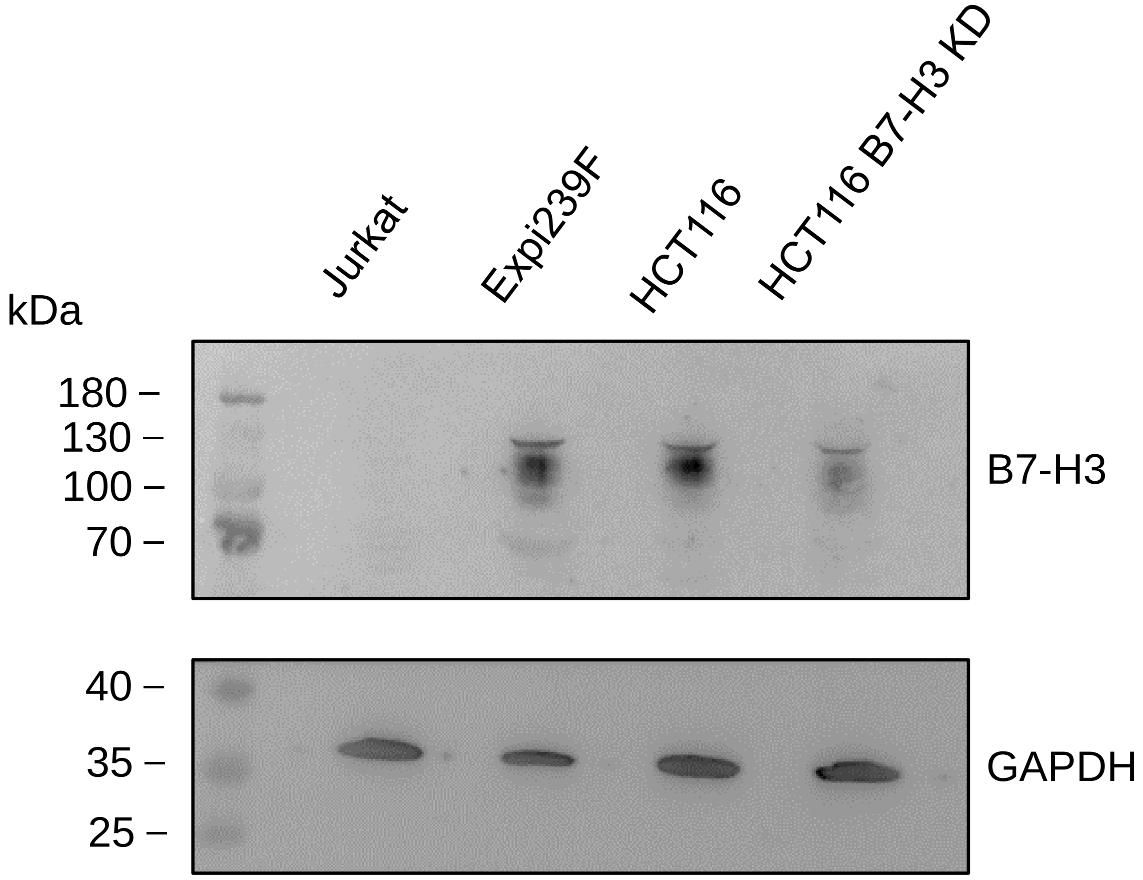

Table S1. Primers for library construction, sanger sequencing, and amplicon preparation for NGS.

Primer name	Sequence (5'→3')
**P1_for**	GCCATGGCCCAGGTGCAGCTGCAGGAAAGCGGCGGCCTGGTGCAGGCGGGCGGCAG
**P2_rev**	GCCGCTCGCCGCGCAGCTCAGGCGCAGGCTGCCGCCCGCCTGC
**P3_for**	CGCGGCGAGCGGCWMTATTTYTNNBNNBNNBNNBATGGGCTGGTATCGCCAGG
**P4_rev**	TTCGCGTTCTTTGCCCGGCGCCTGGCGATACCAGCCCAT
**P5_for**	CCGGGCAAAGAACGCGAAYTTGTTGCCRSTATTRVTNNBGGTRSTANTACCWATTATGCGGATAGCGTG<br>AAAGGCC
**P6_rev**	GTTTTTCGCGTTATCGCGGCTAATGGTAAAGCGGCCTTTCACGCTATCCGCATA
**P7_for**	AGCCGCGATAACGCGAAAAACACCGTGTATCTACAGATGAACAGCCTGAAACC
**P8_rev**	CGCGCAATAATACACCGCGGTATCTTCCGGTTTCAGGCTGTTCATCTGTAGA
**P9a_for**	CCGCGGTGTATTATTGCGCGGYTNNBNNBNNBNNBNNBNNBYWTNNBTATTGGGGCCAGGGCAC<br>C
**P9b_for**	CCGCGGTGTATTATTGCGCGGYTNNBNNBNNBNNBNNBNNBNNBNNBNNBNNBNNBYWTNNBTATT<br>GGGGCCAGGGCACC
**P9c_for**	CCGCGGTGTATTATTGCGCGGYTNNBNNBNNBNNBNNBNNBNNBNNBNNBNNBNNBNNBNNBNNB<br>NNBYWTNNBTATTGGGGCCAGGGCACC
**P10_rev**	GTGGTGTGAGGAGACGGTGACCCCGCTGCTCACGGTCACTTGGGTGCCCTGGCCCCAATA
**MP57-Sequencing-F**	TTTATGCTTCCGGCTCGTATG
**NGS-F**	CTGCGCCTGAGCTGCGCGGCGAGC
**NGS-R**	GCTCACGGTCACTTGGGTGCCCTG
